# Factors associated with medical complications after body art among Israeli adults: a retrospective study

**DOI:** 10.1186/s13584-021-00474-w

**Published:** 2021-09-01

**Authors:** Liat Korn, Hagit Bonny-Noach, Gideon Koren, Rachel Nissanholtz-Gannot

**Affiliations:** 1 Department of Health Systems Management, School of Health Sciences, Ariel University, Ariel, Israel; 2 Department of Criminology, School of Social Sciences, Ariel University, Ariel, Israel; 3 Adelson Faculty of Medicine, Ariel University, Ariel, Israel and Motherisk Israel Program, Shamir Hospital, Be’er Ya’akov, Israel

**Keywords:** Body-art, Tattoos, Piercings, Health complications, Substance use

## Abstract

**Introduction:**

Body-art, including tattoos and piercings, is steadily increasing world-wide but with relatively limited reporting of adverse outcomes. The objective of the present study was to identify correlates that would facilitate a preventative strategy to minimize adverse effects of body-art.

**Methods:**

We examined patterns of body-art, health risk and perceptions among 921 participants (54% female, mean age of 35; SD = 10.8) through in-person questionnaire.

**Results:**

A significantly lower frequency of those with body-art acknowledged that not all venues (parlors, clinics, etc.) are safe in terms of health and hygiene (84.7%t vs. 96.6%, *p < .001*) as compared to those without body-art. Similarly, knowledge of the need for a Ministry of Health certification was reported with lower frequency (77.2% vs. 94.5%, *p < .001*) among those with body-art. Those who experienced medical complications reported higher frequencies of smoking cigarettes and hookah as well as using ecstasy (MDMA). The risk of medical complication after body-art was 4 times higher in those who used ecstasy (OR = 3.97; CI 1.0–14.4; *p < 0.05*). In addition, it was more than 3 times higher for street or home tattooing as compared to studio or a licensed medical center (OR = 3.59; CI 1.32–9.76; *p < .01*), as well as almost 3 times higher among those who did not receive information before performing body-art (OR = 2.70; CI 1.05–6.92; *p < .05*) and who had somebody other than themselves decide on the body-art design (OR = 2.68; CI 1.00–7.19; *p < .05*).

**Conclusions:**

A targeted informational-preventative program should be developed, informed by the risks highlighted in this study. In addition, it would be necessary to draft policies related to regulation and enforcement in order to more effectively manage body-art service provision. The Ministry of Health should supervise and guide tattooists and practitioners regarding the health risks of body-art and offer training and raise awareness among potential clients.

## Introduction

Body-art, also known as body modification, body adornment or body composition [1, 2], is a phenomenon in which a person makes visible changes to their body [3, 4] including tattooing, body piercing of various kinds, subdermal implants in various parts of the body, and surgical modification of bodily features. Studies in various countries have pointed to an increased prevalence of body-art [5–7], particularly among youth [8, 9]. In a 2012 public survey among 2016 Americans [10], 21% reported having one tattoo or more, double the rate in 2008 (14%). Other studies demonstrate that 25% of Americans and up to 47% in certain subpopulations have tattooed themselves [11].

Body-art via piercing, tattooing, and subdermal implants has potential health hazards. Studies have shown that about one-third of people who undergo tattooing develop some complications [12]. Those who undergo body piercing are also susceptible to health risks that derive from the procedure - its location on the body and the clinic where it is done [13]. The most common complications include local infections due to lack of training among those who execute the piercing and deficient hygiene at the clinic. For example, in the oral cavity, piercing may cause accumulation of bacteria and periodontal inflammation. In addition, piercing in the vicinity of the mouth may cause bleeding and nerve damage [14], and may trigger discharge of saliva [15]. Piercing of the tongue may cause gums to recede and, in extreme cases, teeth to fall out. Most pierced people with health concerns return to their body piercers or the Internet for applicable information and nonjudgmental care rather than to medical personnel [16].

In a 2011 study [17] of 1656 students, 78.3% acknowledged the risk of tattooing or piercing, mainly in the forms of HIV-AIDS (60.3%), hepatitis C (38.2%), and tetanus (34.3%). A recent study [18] recommended the development of an explanatory prevention system targeted at young people (especially male teenagers) regarding health and legal risks involving body-art. Presently, identification of at-risk individuals for adverse effects has been sparsely addressed. Any attempts at preventing such risks must identify factors and determinants typical of body-art. The objective of this research was to identify correlates that will facilitate a preventative strategy to minimize adverse body-art effects.

In the present study, we focused on people with body-art and their attendant medical complications. Most of the research on complications of tattoos or piercings focuses on specific medical complications and their prevention [17–21] or the characteristics of the tattoos [22–24] and piercings [20] contributing to the complications. However, in order to prevent medical complications following body-art, risk factors for complications that characterize these subjects need to be identified. As such, this study deals with an important public health hazard and contributes up-to-date data.

## Methods

This is an observational, cross-sectional study aimed at comparing contributing factors for medical complication after body art.

### Procedure

This cross-sectional study was approved by the university’s IRB committee. The participants were recruited by research assistants, who were instructed to approach public places such as malls, train stations and tattooing and piecing venues throughout Israel. The inclusion criteria included adult (18 years or older) Hebrew speakers. A non-probability convenience sampling method **was used** ***in which the sample is taken from a group of people easy to contact or reach*** and was not intended to represent the adult population of Israel. As such, the purpose of this study was to identify factors associated with medical complications of body art and not its prevalence in the population. Hence, research assistants were instructed to approach people who underwent body art in tattooing and piecing venues. In Israel, tattoos are less common than in other developed countries due to religious prohibition. In order to avoid limited sampling of tattooed people, research assistants not only sought participants in public places in different cities in Israel, but also deliberately visited tattoo studios to approach tattooed people. In our case, this method is easily justified because tattoos are considered relatively rare.

### Research sample

The sample comprised 921 participants (53.9% women), with a mean age of 35 (age range: 19–84; SD = 10.8) (Table 1). The participants included married (47.6%), single (39.6%), and divorced, separated or widowed (12.9%) individuals. About half of the sample had children (55.4%) and almost half held an academic degree (48.3%). The sample included 412 subjects (44% of the total) that underwent some form of body-art. Almost a quarter (23.0%) of the sample were 25 years old or younger and more than half (52.6%) were secular. The frequency of reporting body-art was lowest among those with far-above-average income (31.4%).

**Table 1:** Sociodemographic characteristics of the sample and frequency of performing body-art within each subgroup

Variable	Value	Total sample (***N*** = 921)	% of the sample	% of body-art (***n*** = 412)
Gender	Women	496	53.9	52.3
	Men	425	46.1	47.7
Age	19–25	207	23.0	52.2
	26–30	174	19.3	48.3
	31–40	299	33.2	52.5
	41–84	220	24.4	25.9
Religion	Secular	476	52.6	56.9
	Traditional / nonreligious	239	26.4	43.1
	Traditional / religious	104	11.5	19.2
	Religious	67	7.4	11.9
	Other	19	2.0	–
Income	Far above average	51	5.7	31.4
	Above average	233	25.8	48.3
	Similar to average	375	41.6	45.3
	Below average	148	16.4	42.6
	Far below aveage	95	10.5	45.3

### Research tool

This study was based on an original questionnaire which was validated in a pilot study conducted on group of students before delivering it to the whole sample. The questionnaire was structured, self-reported, and covered sociodemographic details, knowledge of medically-related risks associated with body-art, and substances use.

### Description of variables

#### Body-art

This variable refers to participants who have made at least one of the following changes: tattoo, piercing or subcutaneous transplant, based on the question: “Please report which type of body art you have (one or more, not including earlobes).”

#### Medical aspects concerning body-art: knowledge and attitudes

Participants were asked whether they know of any risks concerning body-art (Yes/ No). In the Health and Hygiene Aspects, participants were asked, “Do you think the places (institutes, clinics and else) performing body-art are safe in terms of health and hygiene?” Values were: 1. Yes, all of them; 2. Probably some; 3. Not sure; and 4. Don’t know. Participants were asked if the places performing body-art are approved by the Ministry of Health. Values were: 1. Yes, all of them; 2. Probably some; 3. Not sure; and 4. Don’t know. In addition, participants were asked whether written consent should be signed before undergoing a body-art procedure (Yes/ No/ Don’t know).

#### Obtaining health Hazard information

Participants were asked whether they received information on health risks and complications involved in body art before performing the procedure (Yes/ No). Participants were also asked if they were requested to give written consent prior to the procedure (Yes/ No).

#### Questions to those who underwent body-art

Participants were asked if they suffered from any complications after the procedure (Yes/ No) as well as who made the decision regarding their tattoo design. Values were: 1. Me - this is my original tattoo design; 2. Me - this is a tattoo design that I liked; 3. My spouse; 4. The tattooist; 5. My friends; and 6. I saw this tattoo design on someone else and I liked it; and 7. Other. In addition, another item was: Where was the body-art done? Values were 1. On the street by a street artist; 2. In the tattoo/piercing studio; 3. In a licensed medical center; 4. At the artist’s home; and 5. Other.

#### Substance use

Participants were asked in two different questions if they ever smoked cigaretttes or hookah. Values were: 1. I do not smoke; 2. Less than once a week; 3. At least once a week, but not everyday; and 4. Everyday. Another item asked about binge drinking (as measured by the number of times in the last year five drinks or more were consumed in one event) and drunkness (as measured by number of times the participant was drunk in the last month). Values to both questions ranged from 1- never to 7- more than twice a week. Participants were asked if they used recreational drugs such as cannabis, Ectasy (MDMA), and party drugs, etc. Values were: Any experience (Yes/No) and in the last month (Yes/No).

#### Sociodemographic

Participants were asked about their gender (woman/man) and family status (single, married, divorced/ separated or widowed) and whether they have children.

#### Education

Participants were asked “What is your higher education?”. Values ranged from 1. Highschool-education till 8. Master’s degree and higher. Value 9 was other education.

#### Ethnic origin

Participants were asked “Which ethnic origin / nationality best describes you?” Values were: 1. Ashkenazi / Western Europe; 2. Israeli Arab; 3. Spain / East; 4. Druze, 5. East Europe / Soviet Union / Russia; 6. Bedouin; 7. Ethiopia; 8. Mixed ethnic origins; and 9. Other.

#### Religion

Participants were asked to define their religious orientation. Values were: 1. Secular; 2. Traditional secular; 3. Traditional-religious; 4. Religious; 5. Haredi; and 6. Other: Please specify.

#### Income

Participants were asked to rate their personal income compared to other people their age. Values ranged on a five point Likert scale from 1. well below average to 5. A lot more than average.

### Data analysis

The IBM SPSS Statistics 21 package was used to examine the data and perform the various analyses in this study. Significant differences in rates between subroups was determined by chi-square. Logistic regression was employed to identify significant risk factors related to medical complications of body-art comparing the odds ratios between those who experienced medical complications (study group) and those who did not (control group).

## Results

Of all 921 participants in this study, 405 pariticants (44%) underwent body art. Of those who underwent body art, 71.9% (*n* = 291) got a tattoo, 26.9% a piercing (*n* = 109) and 1.2% (*n* = 5) underwent subcutaneos implantation. Of those with body art, 18% (*n* = 71) reported some type of medical complication (45- infection, 9- trauma, 6- allergic reaction and others such as pain, scars or cysts).

**Fig. 1:**
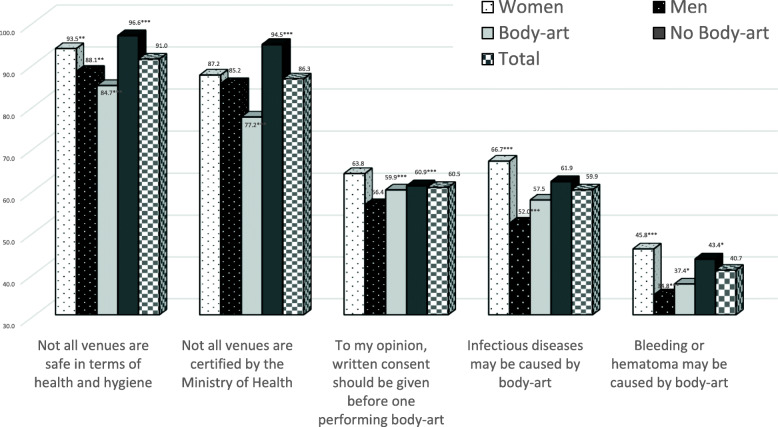
Distribution of health risk knowledge of body-art, by gender and undergoing body-art (%)

The distribution of knowledge of body art medical risk by gender and by whether the participant did or did not undergo body-art is shown in Fig. 1.

Most participants (91.0%) reported being aware that not all venues are medically and hygienically safe or certified by the Ministry of Health. Women reported more frequently than men that not all body-art venues are safe in terms of health and hygiene (93.5% vs. 88.1%, *p < .01*) and they had significantly higher knowledge regarding infectious diseases (66.7 and 45.8% *(p < 0.001)*) as well as body-art caused bleeding or hematoma (52.0 and 34.8%) compared to men. Those with body-art reported lower frequency that not all venues (parlors, clinics, etc.) are safe in terms of health and hygiene (84.7%t vs. 96.6%, *p < .001*) when compared to those without body-art. Similar findings were detected for certification by the Ministry of Health (77.2% vs. 94.5%, *p < .001*). Those with body-art reported less knowledge that bleeding, or hematoma may be caused by body-art (37.4%) compared to those without body-art (43.4%; *p < 0.05*).

Table 2 presents the distribution of substance use frequencies between body-art groups, and complication groups. The table shows significant differences between those who underwent body-art and the non-body-art group in all substance uses, with higher frequencies for those with body-art: For example, cigarettes smoking (56.6% vs. 34.3%; *p* = 0.000), binge drinking (63.6% vs. 38.4%; *p* = 0.000), cannabis (36.8% vs. 20.0%; *p* = 0.000), and party drugs such as Nice Guy (8.3% vs. 3.8%; *p* = 0.005). Significant differences were also found between those who reported medical complications and those who did not among body-art groups in terms of cigarettes smoking (68.3% vs. 55.3%; *p* = 0.040), hookah smoking (46.7% vs. 27.2%; *p* = 0.026), and MDMA (19.0% vs. 8.5%; *p* = 0.014).

**Table 2:** Distribution of substance use frequencies between body-art groups, and complications groups (%)

Variable	Among all sample			Only among body-art group		
	Body-art (*n* = 399 ~ 410)	No body-art (*n* = 472 ~ 502)	*P	Medical complications (*n* = 56 ~ 60)	No medical complications (*n* = 342 ~ 349)	*P
Cigarette smoking (any frequency)	**56.6%**	34.3%	0.000	**68.3%**	55.3%	0.040
Hookah smoking (any frequency)	**30.4%**	17.5%	0.000	**46.7%**	27.2%	0.026
Binge drinking (past year)	**63.6%**	38.4%	0.000	71.7%	64.3%	NS
Drunkenness (past month)	**42.2%**	20.0%	0.000	63.3%	41.8%	NS
Cannabis (any use)	**36.8%**	20.0%	0.000	44.6%	36.0%	NS
Ecstasy (MDMA) (any use)	**9.8%**	3.4%	0.000	**19.0%**	8.5%	0.014
Party drugs Nice Guy (any use)	**8.3%**	3.8%	0.005	**15.8%**	8.2%	0.067
Party drugs Cathinone (any use)	**6.5%**	1.9%	0.001	10.5%	5.8%	NS

*Chi-square significant for differences between independent groups of BM and Medical complications

Table 3 presents the outcome of the logistic regression for association of different study variables with medical complications, among them, sociodemographic variables, substance use and ethical health risk considerations. Four variables had significant relations to medical complications after body-art. The stronger associations with medical complications were the use of MDMA (OR = 3.97; CI 1.0–14.4; *p < 0.05*), the body-art venue (OR = 3.59; CI 1.32–9.76; *p < .01*), receiving information before the procedure (OR = 2.70; CI 1.05–6.92; *p < .05*), and who made the decision regarding the tattoo design (OR = 2.68; CI 1.00–7.19; *p < .05*). The risk of medical complications after body-art was 4 times higher if a participant had used Ecstasy, and more than three times higher for street or home tattoos as compared to a studio or licensed medical center location. The risk was almost 3 times higher among those who did not receive information before, and when somebody else decided the design of the body art.

**Table 3:** Logistic regression to identify risk factors related to medical complications of body-art performance

Variable	Values	OR	95% Confidence Interval for	
			Lower Bound	Upper Bound
Gender	Female = 0, Males = 1	0.81	0.32	2.06
Age (median split)	Older … younger	0.47	0.18	1.23
Religion	Married = 0, Single, Divorced = 1	0.88	0.36	2.15
Income	Yes = 0, No = 1	1.15	0.74	1.79
Ecstasy (MDMA)	Never = 0, Ever = 1	3.97*	**1.09**	**14.4**
Venue of body-art	Studio or a licensed medical center = 0, Street, tattooist home = 1	3.59**	**1.32**	**9.76**
Received information before the procedure	Yes = 0, No = 1	2.70*	**1.05**	**6.92**
Who decided about the tattoo design	Me = 0, All others = 1	2.68*	**1.00**	**7.19**
Cigarette smoking	Never = 0, Ever = 1	1.31	0.50	3.47
Hookah smoking		1.95	0.80	4.73
Binge drinking		1.13	0.41	3.06
Drunkenness		0.73	0.29	1.88
Cannabis		1.75	0.51	5.96
Party drugs- Nice Guy		0.35	0.09	1.33
Party drugs- Cathinone		0.91	0.21	4.01
Written consent before body-art	Yes = 0, No = 1	0.84	0.33	2.15
R^2^ Nagelkerke	23.2%			
N	272			

*p < 0.05, **p < 0.01, ***p < 0.001

## Discussion

This study provides a snapshot of different health risks including knowledge, substance use and ethical aspects concerning medical complications after undergoing body-art. Based on a structured self-reported questionnaire, the results show large differences between participants who underwent body-art and those who did not regarding knowledge of health risks and medical complications as an outcome. Our findings on participant knowledge of health risks and complications indicate scant knowledge among both those who had experience and those who lacked it. Infections and inflammation, although common complications [25, 26], are reported as a possible complication by only 60% of the participants. In addition, only 40% of participants mentioned bleeding or hematoma as possible complications, with women and those who did not undergo any body-art having significantly higher knowledge than men or those who underwent body-art. A survey among students in Italy [17] also revealed partial knowledge regarding medical complications after performing body-art. This point deserves emphasis: it shows that the population that engages in body-art is inadequately aware of the health implications and complications of these procedures. This invokes concern about health ethics in relation to these procedures. It is of importance that level of knowledge of body-art risk should not vary and not directly be associated with perceptions regarding undergoing body-art. Indeed, the desire to get body-art may be stronger than the fear of its complications. In our study, women showed higher knowledge regarding medical complications than men, with those who underwent body-art knowing more about this aspect than those who did not. This may be partially explained by their higher engagement in body-art [17], as showed in our results.

As discussed in the literature, health risk behaviors such as substance use are more common among those who have undergone body-art or those experiencing complications. Tattooed and pierced Israeli individuals reported more smoking, binge drinking and cannabis use [24]. Our findings also include the relations of substance use to medical complications in tattooed and pierced individuals. In our results, participants who experienced medical complications among those who underwent body-art reported more cigarette smoking, hookah smoking and MDMA. In Israel, body-art is much less prevalent than in Europe or North America, possibly due to the prevalence of religious prohibition against body-art.

The differences between those who underwent body-art and those who did not suggest that people who modify their bodies more frequently display interest and curiosity as their initial feelings toward others who have done the same. They also report that they placed more adornments in exposed parts of the body than do people who did not undergo body-art modification. The present findings reveal that the risk of experiencing medical complications after body-art are higher in those who have used MDMA, were tattooed in the street or at the tattooist’s home (rather than in a studio or licensed medical center), did not receive information before the procedure, and whose tattoo design was selected for them. As such, a targeted informational-preventative program should be developed, taking into account the risks highlighted by this study. In addition, policies related to regulation and enforcement should be drafted in order to ensure safer body-art services.

However, this research limitations that should be noted, with one stemming from its convenience sampling method: research assistants were told to approach public places as well as tattoo and piercing venues. That may create a selection bias and can make generalization to the Israeli adult population difficult. Although the main disadvantage of this method is its inability to achieve a measure of prevalence in a population, this was not the purpose of the current research. In fact, non-probability sampling methods were used in studies on tattooing in Italy [17], Malaysia [27], and Texas [28].

Another limitation stems from the self-reported questionnaire which may suffer from wish bias and social desirability. The recommendations expressed above include the development of a focused array of Ministry of Health-derived preventive information strategies oriented at young people (young men in particular) intending to reduce possible body-art health risks. Additional recommendations include drafting relevant policies, along with explicit laws pertaining to body-art – as exist in other countries, as well as closer supervision of these venues. For example, in Italy, a collaborative educational program among body artists was proposed to share information about body-art in general, including inherent risks, encouraging young adults to contemplate their decisions carefully in advance [17]. In Tanzania, registered centers were recommended as well as continued health education and counseling on the risk of complications [19]. Knowledge of body-art complications should be emphasized, not only for health professionals but also the general population [27].

## Conclusions

Significant differences were found between participants who underwent body-art and those who did not regarding knowledge toward health risks and medical complications. This raise concerns that those who engage in body-art are inadequately aware of the medical implications and complications of these procedures. These findings suggest that undergoing body-art during adolescence or young adulthood may create a situation that could spiral out of control in terms of decision-making and, therefore, consequential medical complications. As such, this study advocates for changes in policy: tougher regulation of these venues, with emphasis on compulsory provision of information about complications, and ascertainment that such knowledge is being transmitted and received, along with obtaining informed consent for the procedure. The Ministry of Health should supervise and guide tattooists and practitioners concerning the medical risks of performing body, offering training and raising awareness among potential clients.

## Data Availability

The datasets analyzed during the current study are available from the corresponding author on reasonable request.

## Abbreviations

CI: Confidence interval
IRB: Institutional review board
MDMA: Methylene dioxy meth amphetamine
OR: Odds ratio
SD: Standard deviation
